# Expression of a prostate-associated protein, human glandular kallikrein (hK2), in breast tumours and in normal breast secretions

**DOI:** 10.1054/bjoc.1999.0927

**Published:** 2000-01-18

**Authors:** M H Black, A Magklara, C Obiezu, M A Levesque, D J A Sutherland, D J Tindall, C Y F Young, E R Sauter, E P Diamandis

**Affiliations:** Department of Pathology and Laboratory Medicine, Mount Sinai Hospital, 600 University Avenue, Toronto, Ontario, Canada M5G 1X5 and Department of Laboratory Medicine and Pathobiology, University of Toronto, Toronto, Ontario M5G 1L5, Canada; Toronto Bayview Regional Cancer Centre and Institute of Medical Science, University of Toronto, Toronto, Canada; Department of Urology and Department of Biochemistry and Molecular Biology, Mayo Graduate Schools, Mayo Clinic/Foundation, Rochester, MN 55905, USA; Division of Population Sciences, Fox Chase Cancer Center, Philadelphia, PA 19111, USA

**Keywords:** breast cancer, prostate cancer, human glandular kallikrein, prostate-specific antigen, kallikrein gene family

## Abstract

The recent demonstration of human glandular kallikrein (hK2) expression in a breast carcinoma cell line has suggested that this putatively prostate-restricted, steroid hormone-regulated protease may also be expressed in breast epithelium in vivo and secreted into the mammary duct system. Given that the only substrate yet identified for hK2 activity is the precursor of prostate-specific antigen (PSA), the expression of which in breast carcinomas may be associated with favourable prognosis, our purpose was to examine the expression pattern of both hK2 and PSA in breast tumour tissues. Cytosolic extracts of 336 primary breast carcinomas prepared for routine oestrogen receptor (ER) and progesterone receptor (PR) analysis, as well as 31 nipple aspirates from six women with non-diseased mammary glands, were assayed for hK2 and PSA using immunofluorometric assays developed by the authors. In the tumour extracts, measurable hK2 and PSA concentrations were detected in 53% and 73% of cases respectively, and were positively correlated to each other (*r* = 0.59, *P* = 0.0001). Higher concentrations of PSA and hK2 were found in tumours expressing steroid hormone receptors (*P* = 0.0001 for PSA and *P* = 0.0001 for hK2, by Wilcoxon tests for both ER and PR), and both PSA (*r* = 0.25, *P* = 0.0001) and hK2 (*r* = 0.22, *P* = 0.0001) correlated directly with PR levels. A negative correlation between patient age and PSA (*r* = –0.12, *P* = 0.03) was also found. Both proteins were present in nipple aspirate fluid at relatively high concentrations which were positively correlated (*r* = 0.53, *P* = 0.002). The molecular weights of the immunoreactive species quantified by the hK2 and PSA assays were established by high-performance liquid chromatography (HPLC) and were consistent with the known molecular weights of hK2 and PSA. Together these data provide the first evidence, to our knowledge, that both malignant breast tissue and normal breast secretion contain measurable quantities of hK2, and that the degree of hK2 expression or secretion is directly proportional to the expression of PSA and steroid hormone receptors. hK2 expression may therefore be a marker of steroid hormone action in breast tissue. © 2000 Cancer Research Campaign


*British Journal of Cancer* (2000) **82**(2), 361–367
© 2000 Cancer Research Campaign
Article no. bjoc.1999.0927

### Expression of a prostate-associated protein, human glandular kallikrein (hK2), in breast tumours and in normal breast secretions

**MH Black[1], A Magklara[1], C Obiezu[1], MA Levesque[1], DJA Sutherland[2], DJ Tindall[3], CYF Young[3], ER Sauter[4] and EP Diamandis[1]**

[1]Department of Pathology and Laboratory Medicine, Mount Sinai Hospital, 600 University Avenue, Toronto, Ontario, Canada M5G 1X5 and Department of Laboratory Medicine and Pathobiology, University of Toronto, Toronto, Ontario, Canada M5G 1L5; [2]Toronto Bayview Regional Cancer Centre, and Institute of Medical Science, University of Toronto, Toronto, Canada; [3]Department of Urology and Department of Biochemistry and Molecular Biology, Mayo Graduate Schools, Mayo Clinic/Foundation, Rochester, MN, 55905, USA; [4]Division of Population Sciences, Fox Chase Cancer Center, Philadelphia, PA 19111, USA

**Summary** The recent demonstration of human glandular kallikrein (hK2) expression in a breast carcinoma cell line has suggested that this putatively prostate-restricted, steroid hormone-regulated protease may also be expressed in breast epithelium in vivo and secreted into the mammary duct system. Given that the only substrate yet identified for hK2 activity is the precursor of prostate-specific antigen (PSA), the expression of which in breast carcinomas may be associated with favourable prognosis, our purpose was to examine the expression pattern of both hK2 and PSA in breast tumour tissues. Cytosolic extracts of 336 primary breast carcinomas prepared for routine oestrogen receptor (ER) and progesterone receptor (PR) analysis, as well as 31 nipple aspirates from six women with non-diseased mammary glands, were assayed for hK2 and PSA using immunofluorometric assays developed by the authors. In the tumour extracts, measurable hK2 and PSA concentrations were detected in 53% and 73% of cases respectively, and were positively correlated to each other ($r$ = 0.59, $P$ = 0.0001). Higher concentrations of PSA and hK2 were found in tumours expressing steroid hormone receptors ($P$ = 0.0001 for PSA and $P$ = 0.0001 for hK2, by Wilcoxon tests for both ER and PR), and both PSA ($r$ = 0.25, $P$ = 0.0001) and hK2 ($r$ = 0.22, $P$ = 0.0001) correlated directly with PR levels. A negative correlation between patient age and PSA ($r$ = –0.12, $P$ = 0.03) was also found. Both proteins were present in nipple aspirate fluid at relatively high concentrations which were positively correlated ($r$ = 0.53, $P$ = 0.002). The molecular weights of the immunoreactive species quantified by the hK2 and PSA assays were established by high-performance liquid chromatography (HPLC) and were consistent with the known molecular weights of hK2 and PSA. Together these data provide the first evidence, to our knowledge, that both malignant breast tissue and normal breast secretion contain measurable quantities of hK2, and that the degree of hK2 expression or secretion is directly proportional to the expression of PSA and steroid hormone receptors. hK2 expression may therefore be a marker of steroid hormone action in breast tissue. © 2000 Cancer Research Campaign

**Keywords:** breast cancer; prostate cancer; human glandular kallikrein; prostate-specific antigen; kallikrein gene family

Breast and prostate cancers afflict women and men, respectively, more frequently than any other malignancies. Approximately 40 000 Americans die from each of these cancers every year. Despite the advances in molecular medicine over the last 20 years, our understanding of the pathogenesis of sporadic breast and prostate cancers remains incomplete. Recently, a number of epidemiological, genetic and biochemical similarities between these two hormonally-dependent cancers have been illuminated (Lopez-Otin and Diamandis, 1998), including the expression of proteins initially thought to be restricted to prostatic tissue but now also demonstrated in female breast tissue. The prototype of such proteins is prostate-specific antigen (PSA), which has been shown to be expressed by normal, hyperplastic and cancerous breast epithelium and can be detected in breast secretions including colostrum, nipple aspirate fluid (NAF) and breast cyst fluid (Diamandis and Yu, 1995, 1997). Studies in which several breast cancer cell lines were stimulated with steroid hormones has revealed that expression of PSA mRNA and protein is androgen and progestin-regulated (Zarghami et al, 1997). The role of PSA in breast pathophysiology is unknown, although clues have been provided by observations that its expression is more common in steroid hormone receptor-positive tumours (Diamandis et al, 1994), in smaller, more well-differentiated lesions, and in tumours from younger patients and those who have a favourable clinical prognosis (Yu et al, 1995, 1998).

PSA is a member of the kallikrein family (McCormack et al, 1995), which consists of human glandular kallikrein (hK) 1, hK2 and hK3 (PSA). These genes show extensive sequence homology and localize within a 60 kb region of chromosome 19q13.3–q13.4 (Reigman et al, 1992). Whereas hK1 is widely expressed in various tissues, including kidney and pancreas, expression of both hK2 and hK3 was generally considered prostate-specific until female breast epithelial cells were shown to express and secrete PSA (Diamandis and Yu, 1995, 1997). Extra-prostatic sources of hK2 protein have also been identified in two recent reports, one of which described expression of hK2 by the breast carcinoma cell line T-47D after stimulation by steroid hormones (Hsieh et al, 1997), and another in which the

*Received 26 January 1999*
*Revised 22 June 1999*
*Accepted 22 July 1999*

*Correspondence to:* EP Diamandis presence of hK2 mRNA was demonstrated in human pituitary tumours (Clements et al, 1996).

Recently, work by several groups has suggested the possible parallel expression of PSA and hK2 by prostate and other tissues. hK2, a potent trypsin-like protease, was found able to proteolytically cleave enzymatically inactive pro-PSA into mature, active PSA which has chymotrypsin-like activity (Kumar et al, 1997; Lovgren et al, 1997; Takayama et al, 1997). The co-expression of PSA and hK2 by breast tumours may therefore be based on the ability of pro-PSA, expression of which is hormonally induced, to be activated by the action of co-secreted hK2. We have suggested earlier that enzymatically active PSA may act upon known or unknown substrates to mediate growth-inhibitory or growth-promoting signals (Diamandis, 1996). Among the putative PSA substrates are latent transforming growth factor-β (Killian et al, 1993), insulin-like growth factor binding protein-3 (IGFBP-3) (Cohen et al, 1992), a kinin-like protein (Fichtner et al, 1996) and parathyroid hormone related peptide (Cramer et al, 1996).

Until very recently, the ability to discriminate between PSA and hK2 protein expression has been limited by factors related to their similar structures. In particular, their extensive sequence homology prompted the notion that antibodies raised against either PSA or hK2 will likely cross-react with both antigens. However, monoclonal anti-PSA antibodies free of cross-reactivity to hK2 (Corey et al, 1997), and anti-hK2 monoclonal antibodies free of cross-reactivity to PSA (Saedi et al, 1995; Finlay et al, 1998), have now been developed. The availability of these reagents has, in turn, facilitated the development of highly specific immunoassays for hK2 and PSA (Ferguson et al, 1996; Piironen et al, 1996; Finlay et al, 1998). Evaluation of the cross-reactivities of these antibodies was initially compromised by the inability to isolate either PSA or hK2 from seminal plasma without significant contamination from the other protein. With the recent availability of recombinant hK2 and PSA proteins, this obstacle has also been overcome (Lovgren et al, 1995; Saedi et al, 1995).

In this paper, we present a study which examined the expression levels of both hK2 and PSA in a series of 336 breast tumour cytosolic extracts with respect to each other as well as to their relationships with two clinically important variables – steroid hormone receptors and patient age. The presence of hK2 and PSA in NAF collected from women without apparent breast pathology was also investigated.

### MATERIALS AND METHODS

### Breast tumour cytosolic extracts

This use of human specimens in this study was approved by the Ethics and Research Committee at the University of Toronto. A series of primary breast carcinomas were obtained from 336 females at hospitals collaborating in the Ontario Provincial CEA/Steroid Receptor programme. The breast tumour tissue was snap-frozen in liquid nitrogen immediately after surgical resection, transported as such to the laboratory, and subsequently stored for fewer than 2 weeks at –70°C until extraction was performed. Approximately 0.5 g of tumour tissue was first pulverized at liquid nitrogen temperature, and the resulting powder was combined with 10 ml of extraction buffer (10 mM Tris–HCl, pH 7.40, containing 1.5 mM EDTA and 5 mM sodium molybdate). The suspended tissue powder was solubilized on ice with a single 5 s burst of a Polytron homogenizer (Brinkmann Instruments, Westbury, NY, USA), after which the particulate material was pelleted by centrifugation at 105 000 ***g*** for 1 h. The intermediate layer (cytosolic extract) was collected without disturbing the lipid or particulate layers. Protein concentrations of the cytosolic extracts were determined by the Lowry method. Following steroid hormone receptor quantification, the remainders of the extracts were stored at –70°C for not more than 3 months until PSA and hK2 immunoassay analyses.

### Nipple aspirate fluids

NAFs were obtained from six women, three of which were premenopausal, using the method described in our previous report (Sauter et al, 1996). From each patient, five to six specimens were collected over a 3-month period; 31 NAFs were collected in total. These NAFs, approximately 1–3 μl each, were diluted to 500 μl as described previously (Sauter et al, 1996) and then analysed for PSA, hK2 and total protein.

### Immunochemical assays

For quantitative analyses of ER and PR, enzyme immunoassay kits (Abbott Laboratories, North Chicago, IL, USA) were used according to the manufacturer's instructions. Concentrations of steroid hormone receptors were expressed as fmol of oestrogen receptor (ER) or progesterone receptor (PR) per mg of total protein to adjust for variabilities in specimen masses and extraction efficiencies. The determination of PSA concentrations by time-resolved immunofluorometric assay was performed as described elsewhere (Ferguson et al, 1996). The PSA assay has a detection limit of 1 ng $l^{-1}$ (0.001 μg $l^{-1}$) and has no detectable cross-reactivity to hK2. This assay measures free PSA and PSA bound to its major binding protein, α-1-antichymotrypsin (PSA-ACT), on an equimolar basis. A new time-resolved immunofluorometric assay developed by our group was used to measure hK2 concentrations (Black et al, 1999). The latter assay has a detection limit of 6 ng $l^{-1}$ (0.006 μg $l^{-1}$) and has less than 0.2% cross-reactivity to PSA. Calibration of the PSA assay was performed using highly purified seminal plasma PSA (Ferguson et al, 1996), whereas calibration of the hK2 assay was performed using recombinant hK2 (Black et al, 1999). Results of both immunofluorometric assays were expressed as ng of PSA or hK2 per g of total protein.

### High-performance liquid chromatography

The molecular weights of the immunoreactive species in the breast tumour cytosols were estimated by HPLC using a Superdex 200 gel filtration column (Pharmacia Biotech, Montreal, PQ, Canada) and an HP 1100 series HPLC system (Hewlett-Packard, Mississauga, ON, Canada). The mobile phase consisted of a 50 mM Tris buffer, pH 7.4, containing 0.15 M sodium chloride, which was used at a flow rate of 0.4 ml $min^{-1}$. For each run, a 200 μl volume of tumour cytosol was injected, and 400 μl fractions were collected and assayed for PSA and hK2 as described above. Calibration was achieved by HPLC fractionation under the same conditions of a molecular weight standard solution (BioRad Laboratories, Hercules, CA, USA) containing thyroglobulin ($M_r$ 670 000), IgG ($M_r$ 158 000), ovalbumin ($M_r$ 44 000), myoglobin ($M_r$ 17 000) and cyanocobalamin ($M_r$ 1400).

© 2000 Cancer Research Campaign

**Table 1** Distributions of all numerical variables in 336 breast tumour cytosols

| | PSA (ng g$^{-1}$) | hK2 (ng g$^{-1}$) | PSA/hK2 ratio[a] | ER (fmol mg$^{-1}$) | PR (fmol mg$^{-1}$) | Age (years) |
|---|---|---|---|---|---|---|
| Percentile | | | | | | |
| Minimum | <0.6 | <3.8 | – | 0 | 0 | 29 |
| 5th | <0.6 | <3.8 | – | 0 | 0 | 35 |
| 10th | <0.6 | <3.8 | – | 1 | 1 | 40 |
| 25th | <0.6 | <3.8 | – | 7 | 5 | 47 |
| 50th | 3.7 | 4.2 | 3.2 | 57 | 41 | 57 |
| 75th | 38 | 8.6 | 8.1 | 124 | 216 | 68 |
| 90th | 320 | 29 | 17 | 285 | 410 | 78 |
| 95th | 955 | 79 | 29 | 370 | 502 | 84 |
| Maximum | 400 000 | 7000 | 200 | 980 | 848 | 94 |
| Mean | 718.3 | 28.5 | 9.1 | 99.5 | 132.3 | 58 |
| s.d. | 10 594.3 | 198.2 | 23.6 | 134.3 | 175.6 | 14.6 |
| Number | 336 | 336 | 155 | 336 | 336 | 336 |

[a]Ratio of PSA and hK2 concentrations calculated only for cytosols in which PSA ≥ 0.6 ng g$^{-1}$ and hK2 ≥ 3.8 ng g$^{-1}$.

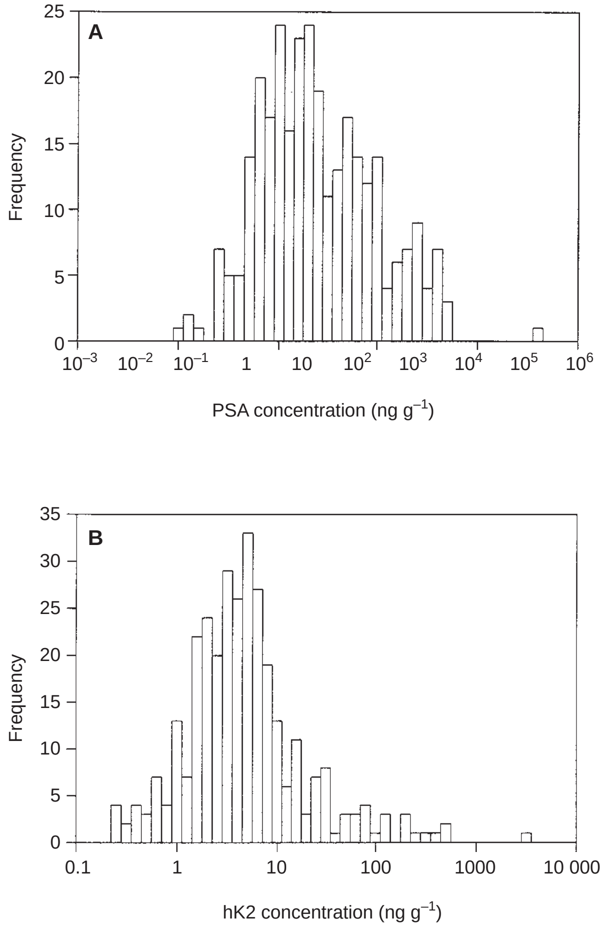

**Figure 1** Frequency distributions of logarithmically-transformed PSA (**A**) and hK2 (**B**) concentrations in 336 breast tumour cytosols

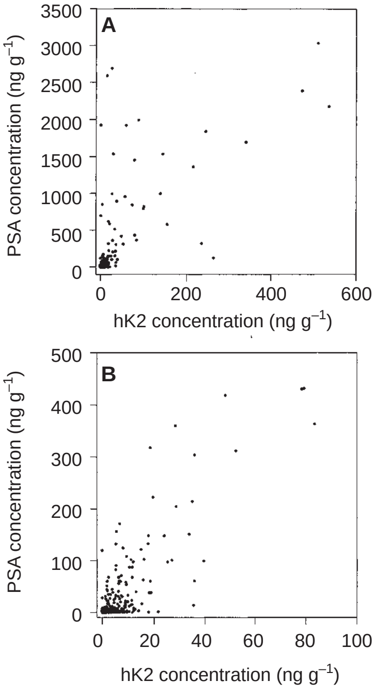

**Figure 2** Scatterplots showing pairs of PSA and hK2 concentrations in: (**A**) 335 breast tumour cytosols, excluding one specimen with a PSA and an hK2 concentration of approximately 400 000 ng g$^{-1}$ and 7000 ng g$^{-1}$, respectively; (**B**) 306 cytosols containing hK2 < 100 ng g$^{-1}$

### Statistical analysis

Associations between PSA, hK2, ER and PR were examined by Spearman correlation and Wilcoxon rank sum tests. Two-sided *P*-values of 0.05 or less were considered statistically significant.

### RESULTS

The total protein concentrations in the breast tumour extracts were normally distributed with a mean of 1.60 g l$^{-1}$ and a standard deviation of 0.5 g l$^{-1}$. Based on the detection limits of the PSA and hK2 immunoassays, total protein-adjusted PSA and hK2 concentrations greater than or equal to 0.6 ng g$^{-1}$ and 3.8 ng g$^{-1}$, respectively, were considered detectable. Among the 336 breast tumour extracts, 73% had detectable PSA and 53% had detectable hK2. The PSA concentrations ranged from 0 to 400 000 ng l$^{-1}$ and were distributed with a median of 5.7 ng l$^{-1}$, a mean of 1408.8 ng l$^{-1}$, and a standard devia-

**Table 2** Wilcoxon rank sum analysis of associations between PSA and hK2 concentrations, steroid hormone receptor status, and patient age

| Variable | Number | Median PSA (ng g$^{-1}$) | *P* | Median hK2 (ng g$^{-1}$) | *P* |
|---|---|---|---|---|---|
| PSA[a] | | | | | |
| PSA (–) | 235 | – | | 0 | |
| PSA (+) | 101 | – | | 15 | <0.001 |
| hK2[b] | | | | | |
| hK2 (–) | 235 | 1.4 | | – | |
| hK2 (+) | 101 | 97 | <0.001 | – | |
| Age | | | | | |
| <57 years | 166 | 5.3 | | 4.4 | |
| >57 years | 170 | 2.9 | 0.06 | 3.9 | 0.24 |
| ER status[c] | | | | | |
| ER (–) | 73 | 0.8 | | 0 | |
| ER (+) | 263 | 5.3 | <0.001 | 5.0 | <0.001 |
| PR status[c] | | | | | |
| PR (–) | 74 | 0.8 | | 0 | |
| PR (+) | 262 | 5.4 | <0.001 | 5.2 | <0.001 |
| Combined ER, PR status[c] | | | | | |
| ER (–), PR (–) | 47 | 0 | | 0 | |
| ER (–), PR (+) | 26 | 5.4 | | 7.0 | |
| ER (+), PR (–) | 27 | 3.4 | | 4.8 | |
| ER (+), PR (+) | 236 | 5.4 | <0.001 | 5.0 | <0.001 |

[a]PSA-positivity based on cut-off level (23 ng g$^{-1}$) equal to the 70th percentile of the PSA distribution. [b]hK2-positivity based on a cut-off level (7 ng g$^{-1}$) equal to the 70th percentile of the hK2 distribution. [c]ER- and PR-positivity based on cut-off levels of 5 fmol mg$^{-1}$.

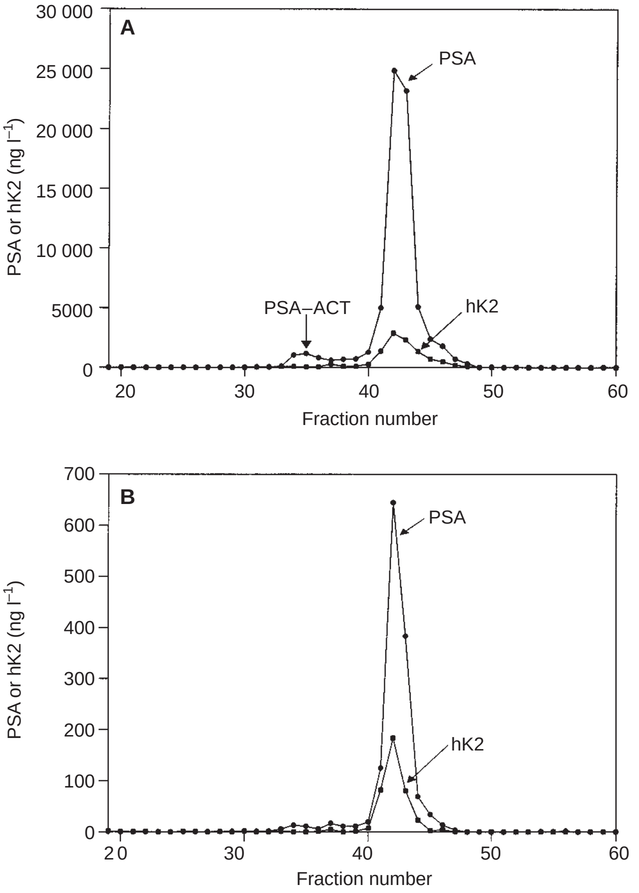

**Figure 3** Gel filtration HPLC chromatograms of two breast tumour cytosols containing approximately: (**A**) 400 000 ng l$^{-1}$ PSA and 7000 ng l$^{-1}$ hK2; (**B**) 3000 ng l$^{-1}$ PSA and 600 ng l$^{-1}$ hK2. The molecular weight markers eluted in fractions 25–26 ($M_r$ 670 000), 32–33 ($M_r$ 158 000), 39–40 ($M_r$ 44 000), 44–45 ($M_r$ 17 000) and 52–53 ($M_r$ 1400)

tion (s.d.) of 21 820.5 ng l$^{-1}$. Concentrations of hK2 ranged from 0 to 7181 ng l$^{-1}$ and had a median, mean, and s.d. of 6.6 ng l$^{-1}$, 48.8 ng l$^{-1}$, and 401.9 ng 1$^{-1}$ respectively. The frequency distributions of all adjusted PSA and hK2 concentrations are shown as histograms in Figure 1 and described in Table 1. Listed also in the table are parameters describing the distributions of ER and PR concentrations, of the PSA/hK2 ratio and of patient age at surgery.

Because the distributions of PSA and hK2 concentrations were positively skewed, indicated by the greatly elevated mean values compared to the respective 50th percentiles, non-parametric statistical procedures using the ranks of the values rather than the values themselves were used to examine relationships between them and the other known parameters. The calculation of Spearman correlation coefficients revealed positive correlation between the pairs of PSA and hK2 concentrations ($r$ = 0.59, $P$ = 0.0001). The wide dispersion of pairs of PSA and hK2 values from a linear relationship is shown in Figure 2. Statistically significant correlations between PSA or hK2 and ER were not found, although positive correlations between PSA ($r$ = 0.25, $P$ = 0.0001) or hK2 ($r$ = 0.22, $P$ = 0.0001) and PR were revealed. There was a moderately strong correlation between ER and PR ($r$ = 0.52, $P$ = 0.0001). Patient age was positively correlated with ER concentration ($r$ = 0.35, $P$ = 0.0001) and negatively correlated with PSA ($r$ = –0.12, $P$ = 0.03), although correlation between age and PR or hK2 were not evident in our series (data not shown).

Categorization of PSA and hK2 concentrations as positive or negative on the basis of cutoff points equal to or greater than the 70th percentiles of the respective distributions, and use of Wilcoxon rank sum tests to compare concentrations of each protein between groups of specimens classified as positive or negative for the other protein, yielded similar findings as the correlation analysis (Table 2). Selection of the cutoff points were arbitrary but consistent with previously used values (Diamandis et al, 1994; Yu et al, 1995). PSA-positive breast tumour cytosols were thus found to have a higher median hK2 level than PSA-negative

© 2000 Cancer Research Campaign

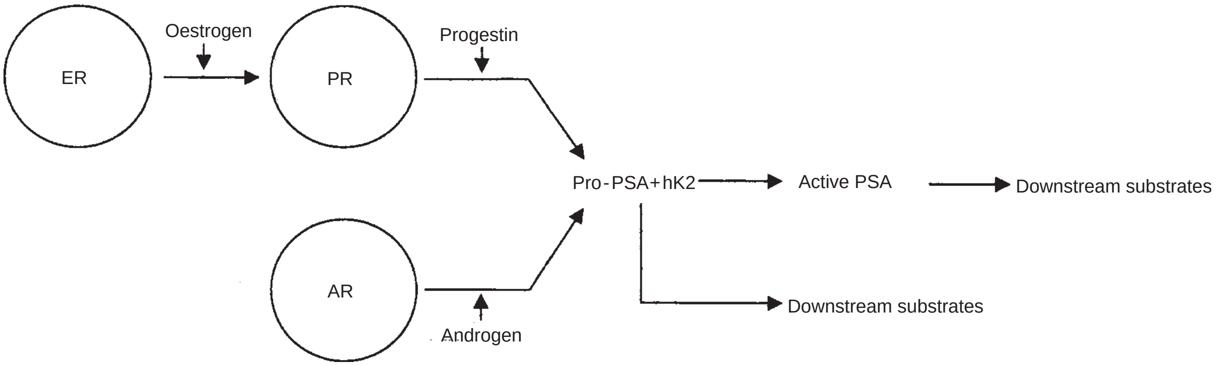

**Figure 4** Hypothetical model of PSA and hK2 regulation in breast tissue. ER, PR and AR are oestrogen, progesterone and androgen receptors respectively. Ligand binding to PR and AR may induce in vivo expression of both pro-PSA and hK2, while ligand binding to ER may up-regulate PR expression. Activation of pro-PSA to mature, enzymatically active PSA may be induced by hK2. Both PSA and hK2 may act on downstream substrates to elicit biological effects specimens. This association was mirrored by the relatively elevated PSA concentrations among hK2-positive tumour extracts compared to hK2-negative specimens. Whereas significant associations between patient age and neither PSA nor hK2 were revealed by this analysis, the increased PSA concentrations in patients less than 57 years of age was of borderline significance. Higher concentrations of both proteins were strongly associated with ER and PR expression, relationships that were evident from comparisons of median PSA or hK2 levels between tumours with negative and positive status for individual receptors, as well as between tumours that were negative for both receptors, positive for PR but negative for ER, positive for ER but negative for PR, and positive for both receptors. The ratio of PSA to hK2 concentrations exceeding the detection limits of the immunoassays varied considerably but, in general, PSA levels were higher by approximately twofold (Table 1).

The molecular weights of the species reactive to the antibodies used in the PSA and hK2 assays were estimated by HPLC in two cytosols containing high contents of these proteins. As shown in Figure 3, the fractions with maximal immunoreactivity in both assays corresponded to a $M_r$ of approximately 30 000. For PSA, a minor fraction (< 5%) which eluted at $M_r$ of approximately 100 000 likely represents PSA bound to the proteinase inhibitor $\alpha_1$-antichymotrypsin, as shown previously (Ferguson et al, 1996).

Despite the necessity to dilute the NAFs by a factor of 100 to 200-fold before analysis (due to their small sample volumes), the measurement of both PSA and hK2 in this fluid was feasible. The median PSA content of the 31 diluted NAFs was 774 ng $g^{-1}$ (range 20–3635 ng $g^{-1}$) and the median hK2 content was 20 ng $g^{-1}$ (range 0–171 ng $g^{-1}$). Only one NAF had undetectable hK2. Spearman analysis between PSA and hK2 concentrations in the NAFs revealed a significant correlation ($r = 0.53$, $P = 0.002$). Based on a total protein content of undiluted NAF of approximately 100 g $l^{-1}$, the median PSA and hK2 concentrations in undiluted NAF were estimated to be approximately 77 000 ng $l^{-1}$ and 2000 ng $l^{-1}$ respectively.

### DISCUSSION

Similarities between two of the most frequently diagnosed human malignancies – breast and prostate cancers – have recently been shown to include the expression of biochemical markers traditionally thought to be specific to prostatic tissue. PSA, currently used in prostate cancer screening and management, has been shown by our group to be present in normal and neoplastic breast epithelium (Diamandis and Yu, 1997) and to be an independent prognostic indicator for breast cancer (Yu et al, 1995, 1998). However, whether the prognostic value of PSA expression is restricted to particular subgroups of breast cancer patients, such as those defined by ER status, lymph node status, or adjuvant treatment remains unclear (Yu et al, 1998). In this report, we present evidence for the expression in breast tissue of another prostate-associated protein, hK2 (McCormack et al, 1995). Although an in vitro study of a breast carcinoma cell line has reported hK2 expression to be up-regulated by steroid hormones (Hsieh et al, 1997), to our knowledge this study is the first in which hK2 expression has been demonstrated in clinical breast tumour specimens. The development of sensitive and specific immunoassays discriminating between the homologous PSA and hK2 proteins (Ferguson et al, 1996; Black et al, 1999), subsequent to the availability of improved antibodies, enabled us to determine the expression pattern of these proteins relative to each other and to patient age and hormone receptor status in a series of newly diagnosed breast carcinomas. Because of the very short post-operative follow-up for these patients, the prognostic implications of hK2 expression compared to that of PSA could not be evaluated.

Of the 336 breast tumour extracts prepared for routine steroid hormone receptor quantification, approximately 70% and 50% contained detectable levels of PSA and hK2 respectively. The median PSA concentration in the tumour extracts was approximately $10^7$–$10^8$ orders of magnitude lower than the level of PSA typically detected in seminal fluid, and about 500-fold lower than serum concentrations of PSA in males, which range from 0 to 4 μg $l^{-1}$ (McCormack et al, 1995). The median hK2 levels in breast tumours are approximately $10^5$-fold lower than that of seminal fluid. Concentrations of both proteins differed widely between specimens although, in general, those of PSA were 3- to 5-fold higher than those of hK2 – a trend previously observed by our group in the sera of males (Black et al, 1999). There is some discrepancy among the reported ratios of circulating PSA and hK2, likely due to differences in assay design and calibration. These inconsistencies are indicative of a need for hK2 assay stan-

© 2000 Cancer Research Campaign dardization, as has long been the case with PSA assays. Throughout the range of PSA and hK2 concentrations, a statistically significant positive correlation was found. We have also established in this series of tumours, as we have in previous cohorts of breast cancer patients (Diamandis et al, 1994; Yu et al, 1998), a negative correlation between PSA expression and patient age. Furthermore, the results of Wilcoxon rank sum tests suggested significantly increased expression of both PSA and hK2 in the presence of ER and PR. While PSA and hK2 concentrations tended to be relatively higher in tumours also expressing ER, we speculate that these latter associations arose indirectly from the strong correlation between ER and PR generally believed to reflect the ability of oestrogen, acting through its receptor, to induce PR expression (McGuire et al, 1977). Support for the notion that PR but not ER is necessary for induction of both PSA and hK2 gene expression has been provided by cell culture studies (Hsieh et al, 1997; Zarghami et al, 1997), as well as the findings reported here of stronger correlations between PSA or hK2 and PR than between either kallikrein and ER. In contrast to the associations between PSA or hK2 and steroid receptor status, the two proteins differed somewhat with respect to their associations with patient age. Younger patients showed a tendency to be more frequently positive for PSA, in accordance with our previously reported data (Diamandis et al, 1994; Yu et al, 1994, 1998), but this relationship was not evident in the case of hK2 expression.

The detection of hK2 in NAF collected from women without apparent malignant disease represents, to our knowledge, a novel finding. Like PSA, which we had previously shown to be present in NAF (Yu et al, 1995, 1996; Foretova et al, 1996; Sauter et al, 1996), hK2 was found at concentrations comparable to those found in seminal plasma. The hK2 concentrations in these 31 specimens were correlated to those of PSA, but were on average about 35 times lower.

The phenomenological data yielded from this study, together with the work of other groups, suggests a possible model for the roles of PSA and hK2 in breast tissue. PSA and hK2 have been shown to be proteases with chymotrypsin-like and trypsin-like activities respectively, and a functional link between these enzymes may be the demonstrated ability of hK2 to proteolytically convert inactive pro-PSA into the enzymatically active, mature PSA which may act upon its physiologically relevant downstream substrates (Kumar et al, 1997; Lovgren et al, 1997). Among the candidate substrates for active PSA is IGFBP-3, cleavage of which liberates the growth factor IGF-1 from its carrier protein (Cohen et al, 1992). PSA may also regulate the activities of other cytokines (Killian et al, 1993; Fichtner et al, 1996), and may itself have growth factor activity (Lai et al, 1996). In addition to PSA, other substrates for hK2 have not yet been identified but may also elicit biological effects. As speculated in Figure 4, the expression of these two enzymes by breast epithelial tissue, and their secretion into mammary ducts, may therefore be directed by common regulatory factors including androgens and progestins. Because the formalin-fixed, paraffin-embedded tissues corresponding to the cytosolic extracts prepared for the breast cancer patients were not available, immunohistochemical determination of the precise cellular source of hK2 could not be performed. It is therefore not presently known if both PSA and hK2 are synthesized by the same cell types in vivo.

In conclusion, we report the presence of hK2 in cytosolic extracts prepared from breast tumours and in secretions of normal mammary tissues, and demonstrate that its expression is correlated to the expression of PSA and to hormone receptor status, particularly that of PR. Because the correlation between concentrations of hK2 and PSA was not perfect, however, it remains possible that hK2 expression may provide prognostic information for breast cancer independently of that provided by PSA or other established indicators of outcome. Future studies will aim to further characterize the hK2–PSA interaction, to determine additional substrates for hK2, to localize PSA and hK2 expression in the context of cytologic features, as well as to address the clinical implications, if any, of hK2 in breast cancer.

### ACKNOWLEDGEMENTS

This work was supported by the Canadian Breast Cancer Research Initiative of the National Cancer Institute of Canada.

### REFERENCES

Black MH, Maglara A, Obiezu C, Melegos DN, Wolfert RL and Diamandis EP (1999) Development of an ultrasensitive immunoassay for human glandular kallikrein (hK2) with no cross reactivity from prostate specific antigen (PSA). *Clin Chem* **45**: 790–799

Clements JA, Mukhtar A, Verity K, Pullar M, McNeill P, Cummins J and Fuller PJ (1996) Kallikrein gene expression in human pituitary tissues. *Clin Endocrinol* **44**: 223–231

Cohen P, Graves HCB, Peehl DM, Kamarei M, Giudice LC and Rosenfeld RG (1992) Prostate-specific antigen (PSA) is an insulin-like growth factor binding protein-3 protease found in seminal plasma. *J Clin Endocrinol Metab* **75**: 1046–1053

Corey E, Buhler KR and Vessella RL (1997) Cross-reactivity of ten anti-prostate-specific antigen monoclonal antibodies with human glandular kallikrein. *Urology* **50**: 567–572

Cramer SD, Chen Z and Peehl DM (1996) Prostate specific antigen cleaves parathyroid hormone-related protein in the PTH-like domain: inactivation of PTHrP-stimulated cAMP accumulation in mouse osteoblasts. *J Urol* **156**: 526–531

Diamandis EP (1996) BRCA1 protein products: antibody specificity, functional motifs and secreted tumour suppressions. *Nat Genet* **13**: 268

Diamandis EP and Yu H (1995) New biological functions of prostate specific antigen? *J Clin Endocrinol Metab* **80**: 1515–1517

Diamandis EP and Yu H (1997) Nonprostatic sources of prostate-specific antigen. *Urol Clin – Am* **24**: 275–282

Diamandis EP, Yu H and Sutherland DJA (1994) Detection of prostate specific antigen immunoreactivity in breast tumors. *Breast Cancer Res Treat* **32**: 301–310

Ferguson RA, Yu H, Kalyvas M, Zammit S and Diamandis EP (1996) Ultrasensitive detection of prostate specific antigen by a time-resolved immunofluorometric assay and the Immulite® Immunochemiluminescent third generation assay: potential applications in prostate and breast cancers. *Clin Chem* **42**: 675–684

Fichtner J, Graves HCB, Thatcher K, Yemoto C and Shortliffe M (1996) Prostate specific antigen releases a kinin-like substance on proteolysis of seminal vesicle fluid that stimulates smooth muscle contraction. *J Urol* **155**: 738–742

Finlay JA, Evans CL, Day JR, Payne JK, Mikolajczyk SD, Millar LS, Kuus-Reichel K, Wolfert RL and Rittenhouse HG (1998) Development of monoclonal antibodies specific for human glandular kallikrein (hK2): development of a dual antibody immunoassay for hK2 with negligible prostate-specific antigen cross-reactivity. *Urology* **51**: 804–809

Foretova L, Garber JE, Sadowsky NL, Verselis SJ and Li FP (1996) Prostate-specific antigen in nipple aspirate. *Lancet* **347**: 1631

Hsieh M, Charlesworth MC, Goodmanson M, Zhang S, Seay T, Klee GG, Tindall DJ and Young CYF (1997) Expression of human prostate-specific glandular kallikrein protein (hK2) in the breast cancer cell line T47-D. *Cancer Res* **57**: 2651–2656

Killian CS, Corral DA, Kawinski E and Constantine RI (1993) Mitogenic response of osteoblast cells to prostate specific antigen suggests an activation of latent TGF-β and a proteolytic modulation of cell adhesion receptors. *Biochem Biophys Res Commun* **192**: 940–947

 © 2000 Cancer Research Campaign

Kumar A, Mikolajczyk SD, Goel AS, Millar LS and Saedi MS (1997) Expression of pro form of prostate-specific antigen by mammalian cells and its conversion to mature, active form by human kallikrein. *Cancer Res* **57**: 3111–3114

Lai LC, Erbas H, Lennard TW and Peaston RT (1996) Prostate-specific antigen in breast cyst fluid: possible role of prostate-specific antigen in hormone-dependent breast cancer. *Int J Cancer* **66**: 743–746

Lopez-Otin C and Diamandis EP (1998) Breast and prostate cancer: common epidemiologic, genetic and biochemical features. *Endocr Rev* **19**: 365–396

Lovgren J, Piironen T, Overmo C, Dowell B, Karp M, Pettersson K, Lilja H and Lundwall A (1995) Production of recombinant PSA and hK2 and analysis of their immunologic cross-reactivity. *Biochem Biophys Res Commun* **213**: 888–895

Lovgren J, Rajakoski K, Karp M, Lundwall A and Lilja H (1997) Activation of the zymogen form of prostate-specific antigen by human kallikrein 2. *Biochem Biophys Res Commun* **238**: 549–555

McCormack RT, Rittenhouse HG, Finlay JA, Sokoloff RL, Wang TJ, Wolfert RL, Lilja H and Oesterling JE (1995) Molecular forms of prostate-specific antigen and the human kallikrein gene family: a new era. *Urology* **45**: 729–744

McGuire WL, Horwitz KB, Pearson OH and Segaloff A (1977) Current status of estrogen and progesterone receptors in breast cancer. *Cancer* **39**: 2934–2947

Piironen T, Lovgren J, Karp M, Eerola R, Lundwall A, Dowell B, Lovgren T, Lilja H and Pettersson K (1996) Immunofluorometric assay for sensitive and specific measurement of human prostate glandular kallikrein (hK2) in serum. *Clin Chem* **42**: 1034–1041

Riegman PH, Vlietstra RJ, Suurmeijer L, Cleutjens CB and Trapman J (1992) Characterization of the human kallikrein locus. *Genomics* **14**: 6–11

Saedi MS, Cass MMJ, Goel AS, Grauer L, Hogen KL, Okaneya T, Griffin BY, Klee GG, Young CY-F and Tindall DJ (1995) Overexpression of a human prostate-specific glandular kallikrein, hK2, in *E. coli* and generation of antibodies. *Mol Cell Endocrinol* **109**: 237–241

Sauter ER, Daly M, Linehan K, Ehya H, Engstrom PF, Bonney G, Ross EA, Yu H and Diamandis EP (1996) Prostate specific antigen levels in nipple aspirate fluid correlate with breast cancer risk. *Cancer Epidemiol Biomarkers Prev* **5**: 967–970

Takayama TK, Fujikawa K and Davie EW (1997) Characterization of the precursor of prostate-specific antigen. *J Biol Chem* **272**: 21582–21588

Yu H, Diamandis EP, Levesque M, Giai M, Roagna R, Ponzone R, Sismondi P, Monne M and Croce C (1996) Prostate specific antigen in breast cancer, benign breast disease and normal breast tissue. *Breast Cancer Res Treat* **40**: 171–178

Yu H, Diamandis EP, Monne M and Croce CM (1995) Oral contraceptive-induced expression of prostate specific antigen in the female breast. *J Biol Chem* **270**: 6615–6618

Yu H, Diamandis EP and Sutherland DJA (1994) Immunoreactive prostate specific antigen levels in female and male breast tumours and its association with steroid hormone receptors and patient age. *Clin Biochem* **27**: 75–79

Yu H, Giai M, Diamandis EP, Katsaros D, Sutherland DJA, Levesque MA, Roagna R, Ponzone R and Sismondi P (1995) Prostate specific antigen is a new favorable prognostic indicator for women with breast cancer. *Cancer Res* **55**: 2104–2110

Yu H, Levesque MA, Clark GM and Diamandis EP (1998) Prognostic value of prostate-specific antigen for women with breast cancer: a large U.S. cohort study. *Clin Cancer Res* **4**: 1489–1497

Zarghami N, Grass L and Diamandis EP (1997) Steroid hormone regulation of prostate specific antigen gene expression in breast cancer. *Br J Cancer* **75**: 579–588

*© 2000 Cancer Research Campaign*
